# Women in low- and middle-income countries receive antenatal care at health institutions, yet not delivered there: a multilevel analysis of 2016–2021 DHS data

**DOI:** 10.1186/s41182-023-00561-5

**Published:** 2024-01-02

**Authors:** Mehari Woldemariam Merid, Dagmawi Chilot, Zeamanuel Anteneh Yigzaw, Alemakef Wagnew Melesse, Menberesibhat Getie Ferede, Fantu Mamo Aragaw, Desalegn Anmut Bitew

**Affiliations:** 1 Department of Epidemiology and Biostatistics, Institute of Public Health, College of Medicine and Health Sciences, University of Gondar, Gondar, Ethiopia; 2 Department of Human Physiology, College of Medicine and Health Sciences, University of Gondar, Gondar, Ethiopia; 3 Department of Health Promotion and Behavioural Sciences, College of Medicine and Health Sciences, Bahir Dar University, Bahir Dar, Ethiopia; 4 Departments of Human Anatomy, College of Medicine and Health Sciences, University of Gondar, Gondar, Ethiopia; 5 Department of Reproductive Health, Institute of Public Health, College of Medicine and Health Sciences, University of Gondar, Gondar, Ethiopia

**Keywords:** Institution delivery dropout, Determinants, Reproductive age women

## Abstract

**Background:**

The institutional delivery dropout (IDD) is a major problem that disproportionately affects low- and middle-income countries (LMICs). It is associated with increased risks of adverse birth outcomes among pregnant women. Hence, this study assessed the pooled estimate and determinants of IDD after antenatal care (ANC) visit among women in LMICs.

**Method:**

The Demographic and Health Survey (DHS) data from 29 LMICs were used for this study. Data analysis was performed with STATA version 14. The forest plot was used to estimate the pooled prevalence of IDD. Multilevel binary logistic regression was fitted to identify determinants of IDD. The statistical significance level between the outcome and independent variables was determined through the adjusted odds ratio (AOR) with 95% CI and p-value less than 0.05.

**Result:**

The pooled prevalence of IDD after ANC booking among reproductive age women in LMICs was 22.25% (95%CI: 18.25, 26.25). Additionally, the prevalence of IDD was highest (29.83%) among women from the South and Central Europe and the Caribbean countries and lowest (13.72%) in Central/Western Asia and the Oceania.

In the multilevel analysis; no education (AOR = 2.92; 95% CI: 2.72, 3.13), poorest wealth index (AOR = 3.46; 95% CI: 3.28, 3.66), inadequate ANC visits (AOR = 1.73; 95% CI: 1.39, 1.77), no media exposure (AOR = 1.27; 95% CI: 1.23, 1.30), rural (AOR = 1.50; 95% CI: 1.43, 1.54), distance a big problem (AOR = 1.28; 95% CI: 1.25, 1.31), and women located in the South/Eastern Europe and Caribbean region 6.67 (AOR = 6.67; 95% CI: 6.20, 7.20), women lived in low-income countries 7.05 (AOR = 7.05; 95% CI: 6.57, 7.56), and women from lower middle-income countries 5.34 (AOR = 5.57; 95% CI: 4.93, 5.78), had increased odds of IDD after ANC among women in LMICs. However, women who had ever born one child (AOR = 0.29; 95% CI: 0.28, 0.31), and women from Central and Western Asia and the Oceania (AOR = 0.78; 95%CI: 0.74, 0.82) had decreased odds of IDD.

**Conclusion:**

The IDD was high among women in LMICs and significantly increased among women with no education, from poorest household, had inadequate ANC visit, no media exposure, rural, distance a big problem. Hence, interventions to reduce IDD should focus on addressing the gaps related to maternal education, access to media, and number of ANC visits among women in LMICs.

## Background

Globally, the maternal morbidity and mortality remained a major public health problem, mostly attributable to preventable causes [1]. Globally, in 2017, it has been estimated that the overall Maternal Mortality Ratio (MMR) was 211 maternal deaths per 100,000 live births where low- and middle-income countries (LMICs) shared disproportionately the highest burden of maternal mortality [2]. As such, the overall estimated MMR in the world’s LMICs in 2017 was 415 maternal deaths per 100 000 live births, more than 40 times higher than that of the developed regions. Particularly, sub-Saharan Africa and South Asia, account for 86 per cent of maternal deaths worldwide [3].

Although pregnant women registered for antenatal care (ANC) follow-up are expected to give birth at health facilities, not maintaining this continuum of care becomes a great challenge in practice [4, 5]. This problem becomes worse in LMICs. For instance, a study conducted in 28 African countries noted that 44% of women were dropped out from institutional delivery after ANC booking [6]. Similarly, studies conducted in 49 countries of the world noted that indicated that the pooled health facility dropout rate was 34% [5].

In LMICs, many deliveries still occur at home where deliveries occur in an unhygienic environment, without a skilled birth attendant and lifesaving medications [3]. Most maternal and neonatal deaths, as well as obstetric complications, have been related to Institutional Delivery Dropout (IDD) in which health workers are unable to reach women’s homes to provide skilled assistance during delivery [7]. In LMICs, direct obstetric complications during childbirth were responsible for 70% of maternal deaths [8]. In addition, pregnant women dropped from health institution delivery could miss a number of maternal and child health care access opportunities. These includes early detection and prompt treatment of delivery related complications, access to immunisation and micronutrient supplementation, information provision on birth preparedness and complication readiness, and related maternal and child health services [9]. Between 2000 and 2017, there has been significant progress in maternal mortality reduction worldwide, declined by 38 percent. However, it remains unacceptably high in LMICs including in sub-Saharan Africa and South Asia regions [3].

Despite the ambitious plan to reduce global maternal mortality ratio to less than 70 per 100 000 live births by 2030, it will be far more difficult to meet this target with the current pace of progress [2, 10]. There is a continued urgent need for maternal health and survival to remain high on the global health and development agenda. It is particularly important that all births are attended by skilled health professionals, as timely management and treatment can make the difference between life and death for the mother as well as for the new-born [10]. In addition, an effective continuum of skilled maternal care ensures that mothers receive essential health packages from pre-pregnancy to delivery and postnatal thereby help to reduce the risk of maternal death.

Although the majority of maternal deaths occurring among women in LMICs is largely due to home deliveries, an aggregate data regarding the magnitude of IDD and its determinants is dearth in LMICs. Some of the previous literatures that assessed the various individual and community level factors affecting IDDs are fragmented and conducted in individual countries and settings which are not aggregate to represent the problem in LMICs [11–25]. Therefore, we aimed to estimate the prevalence of IDD and identify its determinants among reproductive age women booked for ANC follow-up in LMICs using the most recent DHS data.

## Method

### Study population and data source

This study was based on the most recent dataset from the Demographic and Health Survey (DHS) conducted in 29 LMICs between 2016 and 2021. The DHS is a nationally representative, cross-sectional survey that provides reliable data on women, men, and children. It uses the same standardised data collection procedures, sampling, questionnaires, and coding, making the results comparable across countries. Accordingly, the most recent data from the selected LMICs were appended together to estimate the pooled prevalence and identify the determinants of IDD among women of reproductive age group after booked for ANC in LMICs.

The DHS survey employs a two-stage sampling procedure that involves the selection of census enumeration areas from each sampling stratum using a probability proportional to the size of the number of households in each enumeration area in the first stage. In the second stage, households are sampled using systematic random sampling from each enumeration area, which forms the survey clusters.

For this analysis, the study population was women in the reproductive age group who had at least one ANC visit/booked for ANC at a health facility. Between 2016 and 2021, 29 LMICs conducted the DHS survey and about 514, 135 reproductive age women were considered of which a total weighted sample of 335,565 women who had at least one ANC visit at a health facility/booked for ANC were included to the study.

### Study variables and measurement

#### Outcome variable

IDD was considered if women give birth out of health institution after registered for at least one antenatal visit [26].

The outcome variable was generated from the question asked to women who gave birth within 5 years preceding the survey. The response was dichotomised as institutional delivery (if a woman after booked for ANC delivered at any type of health institutions), otherwise categorised as dropout from institutional delivery. Dropout from institutional delivery includes the option given in the survey question termed home of respondents or in others’ home. Health institutions include government hospitals, health centres, health posts, private clinics, or private hospitals. Accordingly, if women deliver at home, we coded “1”, otherwise coded “0”.

#### Independent variables

Based on previous literatures, theoretical and practical significance, both individual and community level variables were included in the study. Accordingly, the variables considered for our study were; the age of respondent, highest educational level attained, marital status, household wealth status, number of ANC visit, total children a woman ever born (parity); categorised as uni-para (a woman ever gave birth of one child), multi-para (2–4 children) grand para (five and above children), pregnancy was wanted when became pregnant, health insurance coverage, media exposure, sex of the household head. On the other hand, place of residence, distance to health facility, countries income status, and world regions were the community level variables considered in this study.

#### Wealth index

In the DHS dataset, wealth index was created using principal components analysis coded as “poorest”, “poorer”, “Middle”, “Richer”, and “Richest and taken as it is.

#### Media exposure

Was generated from women's responses to the questions related to the frequency of listening to the radio, watching television, and reading newspapers per a week. Accordingly, the median of the frequency of listening to the radio, watching television, and reading newspapers was taken and categorised as "yes" if women had above the median value and "no" otherwise.

#### Distance to health facility

It was recorded as a big problem and not a big problem in the dataset was taken without change, which is respondents’ perception during the survey whether they perceived the distance from their home to the nearest health facility to get self-medical help as a big problem or not.

#### Data processing and analysis

We extracted datasets from 29 LMICs’ KR data files and appended them to generate the pooled working data set for the study. Thus STATA version 14 was used to clean, recode and analyse the data. Since the DHS data are hierarchical, i.e. individuals were nested within communities, a multilevel binary logistic regression model was fitted to identify significantly associated factors with IDD among women of reproductive age.

Data analysis was carried out with STATA version 14. Descriptive analysis was carried out using the weighted frequency and percent distribution of the sample for each of the variables. The forest plot was used to explore the pooled prevalence of IDD among the reproductive age women.

To identify determinants of IDD, we used multilevel binary logistic regression because DHS data are hierarchical, i.e. individuals were nested within communities. In this study, the variance inflation factor (VIF) was used to test multi-collinearity and the maximum VIF in this study was 3.08, which indicates the absence of multi-collinearity. Since the models were nested, goodness of model fitness was made using deviance (− 2log-likelihood) where model-III (multilevel) had lowest deviance and hence was the best-fitted model (deviance = 219,443.8).

All variables with a *p*-value ≤ 0.2 in the bi-variable analysis were fitted in the multilevel multivariable model. Adjusted OR (AOR) with 95% CI and *p* < 0.05 were presented to declare statistically significant factors for IDD among pregnant women in LMICs.

## Result

### Description of dropout from institutional delivery by the participants characteristics

In this study, a total weighted 335,565 of women in 29 LMICs were included. Concerning with the age category, 12,182 (21.44%) of the women aged 34–49 years had experienced IDD. By level of education the women attained, larger proportion (30.76%) of the women who had no education were dropped from institutional delivery after registered for ANC. Similarly, only 12.93% of the women who had 5 and above ANC visits during pregnancy had reported IDD. In addition, the proportion of IDD among women who perceived distance to health facility as a big problem was 24.94% **(**Table 1**)**.

**Table 1:** Socio-demographic characteristics of reproductive age women by place of delivery in low- and middle-income countries; (N = 335,565)

Variables	Categories	Place of delivery	
		Out of institution (%)	Institutional (%)
Women’s age in years	15–24	15,975 (15.94)	84,234 (84.06)
	25–34	27,432 (15.36)	151,109 (84.64)
	34–49	12,182 (21.44)	44, 633 (78.56)
Highest educational level	No education	22,849 (30.76)	51,431 (69.24)
	Primary	16,469 (23.96)	52,256 (76.04)
	Secondary	14,821 (10.14)	131,337 (89.86)
	Higher	1450 (3.12)	44,952 (96.88)
Marital status	Currently in union	1397 (14.52)	8,224 (85.48)
	Never in union	51,621 (16.41)	263,001 (83.59)
	Formerly in union	2570 (22.78)	8,751 (77.30)
HH wealth status	Poorest	19,861 (29.13)	48,313 (70.87)
	Poorer	14,561 (20.99)	54,810 (79.01)
	Middle	10,863 (15.87)	57,598(84.13)
	Richer	7215 (10.67)	60,430 (89.33)
	Richest	3089 (4.99)	58,826 (95.01)
Sex of household head	Male	46,223 (16.60)	232,212 (83.40)
	Female	9366 (16.39)	47,764 (83.61)
Total children ever born	Uni-para	9444 (9.50)	89,936 (90.84)
	Multi-para	30,155 (16.16)	156,499 (83.84)
	Grand-para	15,990 (32.28)	33,541 (67.72)
Health insurance covered	Yes	4560 (7.18)	58,917 (92.82)
	No	46,227 (18.40)	204,963 (81.60)
Had media exposure	Yes	31,057 (12.51)	217,228 (87.49)
	No	24,495 (28.10)	62,667 (71.90)
Pregnancy was wanted	Then	44,761 (15.90)	236,820 (84.10)
	Later	6461 (18.39)	28,678 (81.61)
	No more	4360 (23.16)	14,465 (76.84)
Number of ANC visit	1–3	26,462 (23.99)	83,848 (76.01)
	4 and above	29,127 (12.93)	196,128 (87.07)
Community level factors			
Place of residence	Urban	10,134 (8.94)	103,177 (91.06)
	Rural	45,455 (20.45)	176,799 (79.55)
Distance to health facility	No problem	32,126 (13.37)	208,181 (86.63)
	Big problem	23,397 (24.94)	70,421 (75.06)
Region	Sub-Saharan Africa	20,993 (23.79)	67,236 (76.21)
	South and Eastern Europe and the Caribbean	7847 (33.43)	15,627 (66.57)
	Central/western Asia and the Oceania	2440 (12.58)	16,951 (87.42)
	South and Southeast Asia	24,309 (11.89)	180,162 (88.11)
Countries income status	Low income	9523 (22.56)	32,698 (77.44)
	Lower-middle income	40,475 (15.39)	222,542 (84.61)
	Upper-middle income	5590 (18.43)	24,735 (81.57)

### Prevalence of IDD among women of reproductive age in LMICs

**Fig. 1:**
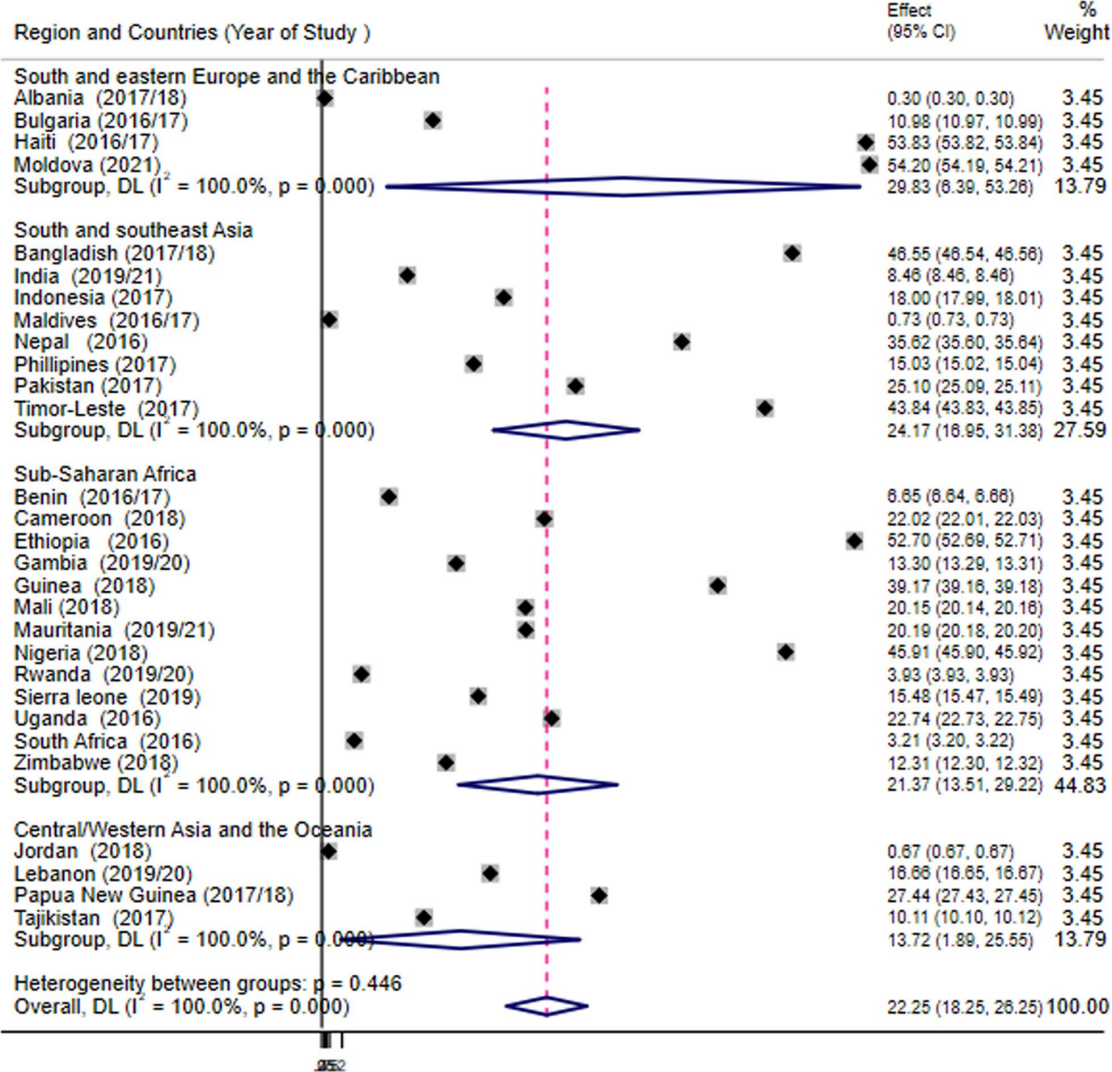
Pooled prevalence of institutional delivery dropout after ANC booking among reproductive age women in low- and middle-income countries; (N = 335,565)

In this study, the pooled prevalence of IDD among reproductive age women in low- and middle-income countries was 22.25% (95%CI: 18.25, 26.25). Additionally, the prevalence of IDD was highest among women from the South and Central Europe and the Caribbean countries [29.83% (95%CI: 6.39 53.26] and lowest in Central/Western Asia and the Oceania [13.72% 95%CI: 1.89, 25.55]. Moreover, the prevalence of IDD was highest in Moldova, 54.20 (95%CI: 54.19, 54.21) (Fig. 1**).**

### Multilevel determinants of institutional delivery dropout

To account for the hierarchical (multilevel) nature of the DHS data, the cluster variable-v001 was considered in the multilevel binary logistic regression. Accordingly, we used multilevel analysis to assess the determinants of IDD among reproductive age women in LMICs. All the variables in the bi-variable analysis had a p-value less than 0.2 and thus fitted to the multivariable analysis.

Accordingly, the odds of IDD among women with no education, primary education, and secondary level of education was 2.92 (95% CI: 2.72, 3.13), 2.35 (95% CI: 2.20, 2.52), and 1.76 (95% CI: 1.65, 1.88), respectively, compared to women who attained higher level of education. Likewise, the household wealth index was also one of the significant determinants of institutional delivery dropout in which women in the poorest household had 3.46 odds of practising IDD compared to those women lived in the richest household (AOR = 3.46; 95% CI: 3.28, 3.66). Also, the odds of IDD was 1.73 (AOR = 1.73; 95% CI: 1.39, 1.77) for women who had inadequate (1–3) ANC visits compared to those who had 4 and above visits.

On the other hand, the odds of IDD among women who had ever born one child was decreased by 61% (AOR = 0.39; 95% CI: 0.37, 0.41) compared to women who had born 4 and above children. Similarly, women who had two to four children had 30% decreased odds of IDD (AOR = 0.70; 95% CI: 0.68, 0.72).

The other variable which significantly determined the prevalence of IDD was the women’s exposure to media. Accordingly, the odds of IDD among women who were exposed to media were 1.27 times higher compared to their counterparts (AOR = 1.27; 95% CI: 1.23, 1.30).

In addition to the above individual level factors; the place of residence, distance to the health facility, region, and income status were the community level factors which determine the prevalence of IDD among women in LMICs.

As such, the odds of IDD among rural women were 1.5 times higher compared to women from urban area (AOR = 1.50; 95% CI: 1.43, 1.54). Also, the odds of IDD was 1.28 (AOR = 1.28; 95% CI: 1.25, 1.31) for women who perceive distance to health facility was a big problem compared to their counterparts. With regard to the region where the women were located, the odds of IDD was 6.67 (AOR = 6.67; 95% CI: 6.20, 7.20) among women from South/Eastern Europe and Caribbean region. On the contrary, women from the Central and western Asia and the Oceania had 20% decreased odds of IDD compared to women from sub-Saharan countries (AOR = 0.80; 95%CI: 0.74, 0.85). Moreover, the odds of IDD among women from LMICs were 7.05 (AOR = 7.05; 95% CI: 6.57, 7.56), and 5.57 (AOR = 5.57; 95% CI: 4.93, 5.78), respectively, compared to women from upper-middle income countries **(**Table 2**).**

**Table 2:** Multi-variable multilevel logistic regression analysis of individual and community level determinants of IDD among reproductive age in LMCs; (N = 335,565)

Variables	Categories	COR [95% CI]	AOR [95% CI]
Women’s age in years	15–24	0.85 (0.82, 0.87)	1.48 (1.42, 1.54)
	25–34	0.86 (0.84, 0.89)	1.19 (1.15, 1.23)
	34–49	Ref	Ref
Highest educational level	No education	11.24 (10.61, 11.91)	**2.92 (2.72, 3.13)**
	Primary	7.37 (6.96, 7.81)	**2.35 (2.20, 2.52)**
	Secondary	3.42 (3.23, 3.62)	**1.76 (1.65, 1.88)**
	Higher	Ref.	Ref.
Marital status	Currently in union	Ref.	Ref.
	Never in union	0.52 (0.49, 0.55)	1.08 (1.01, 1.16)
	Formerly in union	1.05 (1.01, 1.10)	1.12 (1.06, 1.18)
HH Wealth status	Poorest	8.82 (8.45,9.22)	**3.46 (3.28, 3.66)**
	Poorer	5.60 (5.35, 5.8)	**2.56 (2.43, 2.70)**
	Middle	3.88 (3.71,4.05)	**2.09 (1.20, 2.20)**
	Richer	2.37 (2.26, 2.48)	**1.61 (1.53, 1.69)**
	Richest	Ref.	Ref.
Sex of household head	Male	1.14 (1.11, 1.17)	1.20 (1.16, 1.24)
	Female	Ref	Ref
Total children ever born	Uni-para	0.32 (0.31, 0.33)	**0.39(0.37, 0.41)**
	Multi-para	0.57 (0.56, 0.59)	**0.70 (0.68, 0.72)**
	Grand-para	Ref.	Ref.
Health insurance covered	Yes	Ref.	Ref.
	No	2.74 (2.64, 2.84)	1.89 (1.82, 1.97)
Had media exposure	Yes	Ref.	Ref.
	No	2.54 (2.48, 2.59)	**1.27 (1.23, 1.30)**
Pregnancy was wanted	Then	Ref.	Ref.
	Later	0.83 (0.80, 0.85)	0.83 (0.80, 0.86)
	No more	1.25 (1.20, 1.30)	0.95 (0.91, 0.99)
Number of ANC visit	1–3	2.33 (2.28, 2.38)	**1.73 (1.69, 1.77)**
	4 and above	Ref.	Ref.
Place of residence	Urban	Ref.	Ref.
	Rural	3.39 (3.30, 3.48)	**1.50 (1.43, 1.54)**
Distance to health facility	No problem	Ref.	Ref.
	Big problem	1.97 (1.93, 2.01)	**1.28 (1.25, 1.31)**
Region	Sub-Saharan Africa	1.01 (0.98, 1.04)	**0.95 (0.90, 0.98)**
	South and Eastern Europe and the Caribbean	1.55 (1.50, 1.61)	**6.67 (6.20, 7.18)**
	Central/western Asia and the Oceania	0.45 (0.43, 0.47)	**0.80 (0.74, 0.85)**
	South and Southeast Asia	Ref.	Ref.
Countries income status	Low income	1.74 (1.68, 1.80)	**7.05 (6.57, 7.56)**
	Lower-middle income	1.32 (1.27, 1.37)	**5.34 (4.93, 5.78)**
	Upper-middle income	Ref.	Ref.

*COR* crude odds ratio, *AOR*  adjusted odds ratio, CI confidence interval*,* ref. *reference*

## Discussion

Maternal mortality is a serious global public health problem, considerably affecting women in resource-limited countries, mainly women who practice delivery out of health institutions. The current study assessed the pooled prevalence of IDD and its determinants after ANC booking among reproductive age women in LMICs using the DHS data conducted between 2016 and 2021.

In this study, the pooled prevalence of IDD after ANC booking among women in LMICs was 22.25%. This finding was lower compared to two pooled estimates of IDD of which one has been conducted among women in 28 African countries [6] and the other among women across 49 countries in LMICs [27]. Also, the prevalence of IDD in our study was lower compared to the previous studies conducted in different countries [28–33]. The observed differences in the magnitude of IDD between our finding and others could be attributed to the inequalities in terms of countries wealth status, differences in the distributions of maternal and child health services across nations, and due to the diverse socio-cultural factors across the LMICs which actually lead women opt to drop from institutional delivery [34–37].

In the current study, we also assessed different socio-demographic, economic, and reproductive related variables at the individual and community levels which could have influence on IDD after women have booked for ANC in LMICs. As expected, the IDD was higher among women who had inadequate ANC visits compared to those who had four and above visits in our study. A number of studies consistently reported that ANC utilisation was an important determinant of choice of place of delivery [12, 14, 20, 38, 39]. Often times, it is supposed that women at their contacts during ANC visits will obtain adequate information regarding their pregnancy status and possible complications, which they may encounter during childbirth and the possible management options available at the health facilities [40, 41]. Besides, women who have adequate ANC visits would have frequent contacts with the healthcare providers and they may develop trust and confidence to prefer delivery at the health facilities [42].

In our study, the odds of IDD was 2.92 (AOR = 2.92; 95% CI: 2.72, 3.13) times higher among women with no education compared to women who attained higher level of education. Likewise, women who attained primary level of education had 2.35 odds of IDD **(**AOR = 2.35; 95% CI: 2.20, 2.52**).** In line with our finding, previous studies elsewhere noted a positive correlation in that the higher the level of women’s education, the more likely they prefer to give birth at health institutions and thus the less likely to experience ID [13, 16, 20, 22, 43–45]. The association could be explained in that the more educated a woman could easily understand the possible complications of pregnancy and childbirth and thus able to make better choices for herself and her baby [46–48].

In this study, we found that the odds of IDD among women who had ever born one child was decreased by 71% (AOR = 0.29; 95% CI: 0.28, 0.31) compared to women who had born five and above children. Similar results were reported by studies elsewhere where a lower prevalence of IDD was found among women who gave birth of one child [19, 23, 24, 41, 49]. This can be explained in that women who gave only one birth could not have many experiences of childbirth and are more concerned about their delivery thus they are more likely to deliver at health facility compared to the multiparous women [43, 50]. Besides, women with sufficient past delivery experiences may consider care during the previous deliveries as less relevant [6]. Hence, women with higher parity in LMICs should be targeted during educational intervention programmes to raise their awareness regarding the importance of deliveries in the health facilities.

In this study, the household wealth index and countries level of income were the key socio-economic determinants of IDD among women in LMICs. As such, we found that women who lived in the poorest wealth quintile household had 3.46 higher odds of IDD compared to those women lived in the richest quintile. Likewise, the odds of IDD among women from LMICs were 7.05 and 5.57, respectively, compared to women from upper-middle income countries. Such strong association between wealth/income and IDD was consistent with previous studies from different LMICs [13, 14, 16, 21, 22, 25, 41, 44, 51]. The observed wealth–health correlation in terms of the choice of place of delivery among the rich and the poor could possibly be attributed to financial need to access and use healthcare resources in general. As such, poor women might have financial hardships including the cost of transport and buying other items required for delivery when there is the need to deliver in a health facility [19, 52]. It is therefore highly recommended that intervention strategies in countries need to consider such wealth inequalities as a proximal predictor to the choice of place of delivery.

In this study, the odds of IDD were higher among women who had no media exposure compared to their counterparts. This finding was parallel to different studies conducted elsewhere in the world [16, 51, 53, 54]. Indeed, exposure to media is one of the proximal determinants for healthcare information access including the information relevant to the choice of place of delivery. This can be justified in that most media programmes might promote health facility delivery, which may influence women to develop a positive behaviour towards facility delivery [55].

Another factor that showed an important influence on IDD was women’s place of residence. As such, the odds of IDD among rural women were 1.5 times higher compared to women from urban area (AOR = 1.50; 95% CI: 1.43, 1.54). In line with this, living in rural area was associated with higher odds of IDDs in different countries [15–17, 56]. This can be attributed mainly to the obvious reason that hard to reach areas, mostly rural locations are discriminately denied of the healthcare access including maternal and child health services. Women in remote areas are also challenged by transport difficulties in reaching health facilities and lack adequate infrastructure for care [24, 57].

Similarly, the odds of IDD was 1.28 (AOR = 1.28; 95% CI: 1.25, 1.31) for women who perceived distance to health facility was a big problem compared to their counterparts. This relationship was supported with studies elsewhere [14, 19, 21]. The association could be explained in that long distance to health institutions is a real barrier to accessing care [58]. For most pregnant women in LMICs, it is hard to walk several kilometres to seek care for maternity care and even impossible if labour begins at night when transport is often unavailable [58, 59]. The barrier effect of the distance is the strongest when combined with the lack of transport, financial hardships, and poor road conditions [12].

With regard to the region where the women were located, the odds of IDD was 6.67 (AOR = 6.67; 95% CI: 6.20, 6.70) among women from South/Eastern Europe and Caribbean region. On the contrary, women from the Central and western Asia and the Oceania had 20% decreased odds of IDD compared to women from sub-Saharan countries (AOR = 0.80; 95%CI: 0.74, 0.85). Based on the WHO evaluation of sub-regions progress towards SDG target, the Central and Western Asia roughly halved their MMRs mainly due to an aggressive efforts of providing comprehensive maternity services including the enhanced institutional deliveries [2]. However, women in South/Eastern Europe and Caribbean region were more likely to experience IDD partly due to countries in these sub-regions encouraged home delivery for women with low-risk, under the supervision of a skilled birth attendants [37].

There are some noteworthy limitations to mention of this study. Firstly, due to the cross-sectional nature of the data collected, despite the strong and significant associations between variables, it cannot be a guarantee of causality of the associations. The second limitation is that the recall and self-reporting bias might have been introduced. Lastly, the health facility readiness variable, an important determinant of IDD, was unavailable in the dataset we analysed. Hence, we acknowledged this limitation and kindly recommend future researchers to incorporate this variable as a key determinant of IDD among women in LMICs.

## Conclusion

The rate of IDD was high among women in LMICs mainly driven by socio-economic problems such as no or low-level education, poverty, and problem on distance to health facility. Hence, tailored and appropriate interventions to reduce IDD should focus on addressing inequities associated with family wealth, maternal education, access to the media, and number of ANC visit, which ultimately would help narrowing the gap between rural and urban areas, educated and uneducated women, and poor and rich families.

## Data Availability

Data are available online in a public, open-access repository (www.measuredhs.com/data).

## Abbreviations

ANC: Antenatal care
AOR: Adjusted odds ratio
DHSs: Demographic and Health Surveys
ICC: Intra-cluster correlation coefficient
LMICs: Low- and middle-income countries
MOR: Median odds ratio
MMR: Maternal mortality ration
PCV: A proportional change in variance
KR: Kids record
SDG: Sustainable Development Goal
WHO: World Health Organization

## References

[1] Ozimek JA, Kilpatrick SJ (2018). Maternal mortality in the twenty-first century. Obstet Gynecol Clin.

[2] World Health Organization (2019). Trends in maternal mortality 2000 to 2017: estimates by WHO, UNICEF, UNFPA, World Bank Group and the United Nations Population Division, executive summary.

[3] NUICEF. Monitoring the situation of children and women. 2023. https://data.unicef.org/topic/maternal-health/maternal-mortality/. Accessed 15 Feb 2023.

[4] Jolivet RR (2018). Ending preventable maternal mortality: phase II of a multi-step process to develop a monitoring framework, 2016–2030. BMC Pregnancy Childbirth.

[5] Rammohan A (2021). Exposure to conflicts and the continuum of maternal healthcare: analyses of pooled cross-sectional data for 452,192 women across 49 countries and 82 surveys. PLoS Med.

[6] Chukwuma A (2017). Quality of antenatal care predicts retention in skilled birth attendance: a multilevel analysis of 28 African countries. BMC Pregnancy Childbirth.

[7] Newborns, W., *Improving survival and well-being.* World Health Organization. 2020. https://www.who.int/news-room/fact-sheets/detail/newborns-reducing-mortality.

[8] Ronsmans C (2002). Questioning the indicators of need for obstetric care. Bull World Health Organ.

[9] Kerber KJ (2007). Continuum of care for maternal, newborn, and child health: from slogan to service delivery. The Lancet.

[10] World Health Organization. SDG Target 3.1 Maternal mortality. https://www.who.int/data/gho/data/themes/topics/sdg-target-3-1-maternal-mortality. Accessed 15 Feb 2023.

[11] Tsegay R (2017). Determinant factors of home delivery among women in Northern Ethiopia: a case control study. BMC Public Health.

[12] Okeshola FB, Sadiq IT (2013). Determinants of home delivery among Hausa in Kaduna south local government area of Kaduna state, Nigeria. Am Int J Contemp Res.

[13] Kimario FF (2020). Determinants of home delivery among women aged 15–24 years in Tanzania. Int J Matern Child Health AIDS.

[14] Gebremichael SG, Fenta SM (2021). Determinants of institutional delivery in Sub-Saharan Africa: findings from Demographic and Health Survey (2013–2017) from nine countries. Trop Med Health.

[15] Gurung MS (2018). Factors associated with delivery at home in Bhutan: findings from the National Health Survey 2012. WHO South East Asia J Public Health.

[16] Bado AR (2022). Factors associated with home births in Benin and Mali: evidence from the recent demographic and health surveys. Front Reprod Health.

[17] Hernández-Vásquez A (2021). Factors associated with home births in Peru 2015–2017: a cross-sectional population-based study. Heliyon.

[18] Lukonga E, Michelo C (2015). Factors associated with neonatal mortality in the general population: evidence from the 2007 Zambia Demographic and Health Survey (ZDHS); a cross sectional study. Pan Afr Med J.

[19] Kitui J, Lewis S, Davey G (2013). Factors influencing place of delivery for women in Kenya: an analysis of the Kenya demographic and health survey, 2008/2009. BMC Pregnancy Childbirth.

[20] Banke-Thomas OE, Banke-Thomas AO, Ameh CA (2017). Factors influencing utilisation of maternal health services by adolescent mothers in Low-and middle-income countries: a systematic review. BMC Pregnancy Childbirth.

[21] Tessema ZT, Tesema GA (2020). Pooled prevalence and determinants of skilled birth attendant delivery in East Africa countries: a multilevel analysis of Demographic and Health Surveys. Ital J Pediatr.

[22] Budu E (2020). Predictors of home births among rural women in Ghana: analysis of data from the 2014 Ghana Demographic and Health Survey. BMC Pregnancy Childbirth.

[23] Maximore LS (2022). Prevalence and determinants of home delivery among reproductive age women, Margibi County, Liberia. BMC Pregnancy Childbirth.

[24] Amit AML (2022). Prevalence and determinants of home delivery in urban and rural Philippines: Evidence from the 2017 National Demographic and Health Survey. Womens Health (Lond).

[25] Tessema ZT, Tiruneh SA (2020). Spatio-temporal distribution and associated factors of home delivery in Ethiopia. Further multilevel and spatial analysis of Ethiopian demographic and health surveys 2005–2016. BMC Pregnancy Childbirth.

[26] Muluneh AG (2020). High dropout rate from maternity continuum of care after antenatal care booking and its associated factors among reproductive age women in Ethiopia, evidence from Demographic and Health Survey 2016. PLoS ONE.

[27] Akinyemi OJ, Adebowale AS, Rammohan A (2021). Exposure to conflicts and the continuum of maternal healthcare: analyses of pooled cross-sectional data for 452,192 women across 49 countries and 82 surveys. BMC Pregnancy Childbirth.

[28] Tariku M (2022). More than one-third of pregnant women in Ethiopia had dropped out from their ANC follow-up: evidence from the 2019 Ethiopia mini demographic and health survey. Front Glob Womens Health.

[29] Abebe GF (2022). Multilevel analysis of the predictors of completion of the continuum of maternity care in Ethiopia; using the recent 2019 Ethiopia mini demographic and health survey. BMC Pregnancy Childbirth.

[30] Akinyemi JO, Afolabi RF, Awolude OA (2016). Patterns and determinants of dropout from maternity care continuum in Nigeria. BMC Pregnancy Childbirth.

[31] Gandhi S (2022). Predictors of the utilisation of continuum of maternal health care services in India. BMC Health Serv Res.

[32] Mihret MS (2022). Risk factors of dropout from institutional delivery among HIV positive antenatal care booked mothers within one year postpartum in Ethiopia: a case-control study. Arch Public Health.

[33] Yalew M (2023). Spatial distribution and associated factors of dropout from health facility delivery after antenatal booking in Ethiopia: a multilevel analysis. Arch Public Health.

[34] Atake EH (2021). Socio-economic inequality in maternal health care utilization in Sub-Saharan Africa: evidence from Togo. Int J Health Plann Manag.

[35] Deogaonkar M (2004). Socio-economic inequality and its effect on healthcare delivery in India: inequality and healthcare. Electron J Sociol.

[36] Adeyanju O, Tubeuf S, Ensor T (2017). Socio-economic inequalities in access to maternal and child healthcare in Nigeria: changes over time and decomposition analysis. Health Policy Plan.

[37] Joseph G (2016). Inequalities in the coverage of place of delivery and skilled birth attendance: analyses of cross-sectional surveys in 80 low and middle-income countries. Reprod Health.

[38] Pandey AR (2017). Determinants of home delivery among women attending antenatal care in Bagwai Town. Kano Nigeria PLoS One.

[39] Sadia A (2022). Factors associated with home delivery in rural Sindh, Pakistan: results from the global network birth registry. PLoS ONE.

[40] Sadia A (2022). Factors associated with home delivery in rural Sindh, Pakistan: results from the global network birth registry. BMC Pregnancy Childbirth.

[41] Dahiru T, Oche OM (2015). Determinants of antenatal care, institutional delivery and postnatal care services utilization in Nigeria. Pan Afr Med J.

[42] Boah M, Mahama AB, Ayamga EA (2018). They receive antenatal care in health facilities, yet do not deliver there: predictors of health facility delivery by women in rural Ghana. BMC Pregnancy Childbirth.

[43] Gebregziabher NK (2019). Factors determining choice of place of delivery: analytical cross-sectional study of mothers in Akordet town, Eritrea. BMC Public Health.

[44] Hernández-Vásquez A, Chacón-Torrico H, Bendezu-Quispe G (2021). Prevalence of home birth among 880,345 women in 67 low-and middle-income countries: a meta-analysis of demographic and health surveys. SSM-population Health.

[45] Were LP (2017). The Association of Health Insurance with institutional delivery and access to skilled birth attendants: evidence from the Kenya demographic and health survey 2008–09. BMC Health Serv Res.

[46] Belay A, Sendo E (2016). Factors determining choice of delivery place among women of child bearing age in Dega Damot District, North West of Ethiopia: a community based cross- sectional study. BMC Pregnancy Childbirth.

[47] Ayalew HG (2022). Spatial variation and factors associated with home delivery after ANC visit in Ethiopia; spatial and multilevel analysis. PLoS ONE.

[48] Ganle JK (2019). Understanding factors influencing home delivery in the context of user-fee abolition in Northern Ghana: evidence from 2014 DHS. Int J Health Plann Manage.

[49] Dhakal PM (2018). Factors affecting the place of delivery among mothers residing in Jhorahat VDC, Morang, Nepal. Int J Community Based Nurs Midwifery.

[50] Ekele BA, Tunau KA (2007). Place of delivery among women who had antenatal care in a teaching hospital. Acta Obstet Gynecol Scand.

[51] Gudayu TW (2022). Determinants of place birth: a multinomial logistic regression and spatial analysis of the Ethiopian mini demographic and health survey data, 2019. BMC Pregnancy Childbirth.

[52] Wagle RR, Sabroe S, Nielsen BB (2004). Socioeconomic and physical distance to the maternity hospital as predictors for place of delivery: an observation study from Nepal. BMC Pregnancy Childbirth.

[53] Alemayehu M, Mekonnen W (2015). The prevalence of skilled birth attendant utilization and its correlates in North West Ethiopia. Biomed Res Int.

[54] Devkota B, Maskey J (2020). Determinants of home delivery in Nepal—a disaggregated analysis of marginalised and non-marginalised women from the 2016 Nepal Demographic and Health Survey. PLoS ONE.

[55] Yebyo H, Alemayehu M, Kahsay A (2015). Why do women deliver at home? Multilevel modeling of Ethiopian National Demographic and Health Survey data. PLoS ONE.

[56] Solanke BL, Rahman SA (2018). Multilevel analysis of factors associated with assistance during delivery in rural Nigeria: implications for reducing rural-urban inequity in skilled care at delivery. BMC Pregnancy Childbirth.

[57] Health, N.F.M.o., Saving newborn lives in Nigeria: Newborn health in the context of the Integrated Maternal, Newborn and Child Health Strategy. Federal Ministry of Health. 2011

[58] Thaddeus S, Maine D (1994). Too far to walk: maternal mortality in context. Soc Sci Med.

[59] Weeks A (2015). The prevention and treatment of postpartum haemorrhage: what do we know, and where do we go to next?. BJOG.

